# DNA Display Selection of Peptide Ligands for a Full-Length Human G Protein-Coupled Receptor on CHO-K1 Cells

**DOI:** 10.1371/journal.pone.0030084

**Published:** 2012-01-10

**Authors:** Nobuhide Doi, Natsuko Yamakawa, Hideaki Matsumoto, Yasutsugu Yamamoto, Tetsuya Nagano, Nobutaka Matsumura, Kenichi Horisawa, Hiroshi Yanagawa

**Affiliations:** Department of Biosciences and Informatics, Keio University, Yokohama, Japan; Consejo Superior de Investigaciones Cientificas, Spain

## Abstract

The G protein-coupled receptors (GPCRs), which form the largest group of transmembrane proteins involved in signal transduction, are major targets of currently available drugs. Thus, the search for cognate and surrogate peptide ligands for GPCRs is of both basic and therapeutic interest. Here we describe the application of an *in vitro* DNA display technology to screening libraries of peptide ligands for full-length GPCRs expressed on whole cells. We used human angiotensin II (Ang II) type-1 receptor (hAT1R) as a model GPCR. Under improved selection conditions using hAT1R-expressing Chinese hamster ovary (CHO)-K1 cells as bait, we confirmed that Ang II gene could be enriched more than 10,000-fold after four rounds of selection. Further, we successfully selected diverse Ang II-like peptides from randomized peptide libraries. The results provide more precise information on the sequence-function relationships of hAT1R ligands than can be obtained by conventional alanine-scanning mutagenesis. Completely *in vitro* DNA display can overcome the limitations of current display technologies and is expected to prove widely useful for screening diverse libraries of mutant peptide and protein ligands for receptors that can be expressed functionally on the surface of CHO-K1 cells.

## Introduction

The superfamily of G protein-coupled receptors (GPCRs) [1] is the largest and most diverse group of cell-surface proteins involved in signal transmission. Although a large number of GPCRs has been identified in the human genome project [2], [3], more than 100 of them have no known physiologically relevant ligand yet [4], [5], and they are classified as orphan GPCRs. Since GPCRs are major targets for today's drugs [6], the search for cognate and surrogate peptide ligands for GPCRs is of both basic and therapeutic interest [7], [8]. Conventional analysis of the specificity of the interaction between GPCRs and peptide ligands involves the mutation of individual amino acids by peptide synthetic methods (*e.g.*, Ala-scanning), followed by measurement of binding affinity or receptor activation. However, the sequence space that can be searched with this standard strategy is quite limited.

As a powerful alternative strategy, phage display has been used to screen peptides that bind to GPCRs expressed on mammalian cells [9]–[13], but the library sizes and the sequence varieties in a phage library are limited by the transformation efficiency and biological constraints of the host bacteria. This limitation can potentially be overcome by using totally *in vitro* selection systems, such as ribosome display and mRNA display, which employ cell-free protein synthesis [14]–[17]. Recently, mRNA display was used to screen peptide ligands that bind to the N-terminal extracellular domain of a class B GPCR immobilized on beads [18], but such an RNA-tagging method requires strictly RNase-free conditions and cannot easily be applied to selection targeting full-length GPCRs expressed on the cell surface.

We have previously developed a DNA display system called STABLE (STreptAvidin-Biotin Linkage in Emulsions) [19]–[21], in which streptavidin-fused peptides are linked with their encoding DNA *via* biotin labels in a cell-free transcription/translation system compartmentalized in water-in-oil emulsions. This method allows completely *in vitro* selection of a stable DNA-tagged peptide library with large diversity in the presence of RNase. In this study, we applied the DNA display system to *in vitro* selection of peptide ligands for a full-length GPCR expressed on whole cells. As a model to test our screening strategy, we used a well-known GPCR, human angiotensin II (Ang II) type-1 receptor (hAT1R), which is significantly involved in cardiovascular diseases. Under improved selection conditions using hAT1R-expressing mammalian cells as bait, the Ang II gene was enriched from model libraries (1∶100 or 1∶10,000 mixture of streptavidin-fused Ang II and streptavidin genes). Further, various Ang II-like peptides were successfully selected from randomized peptide libraries, and their binding activity and biological function were characterized to elucidate the sequence-function relationship of hAT1R ligands.

## Results

### Strategy for *in vitro* selection of GPCR-ligands

**Figure 1:**
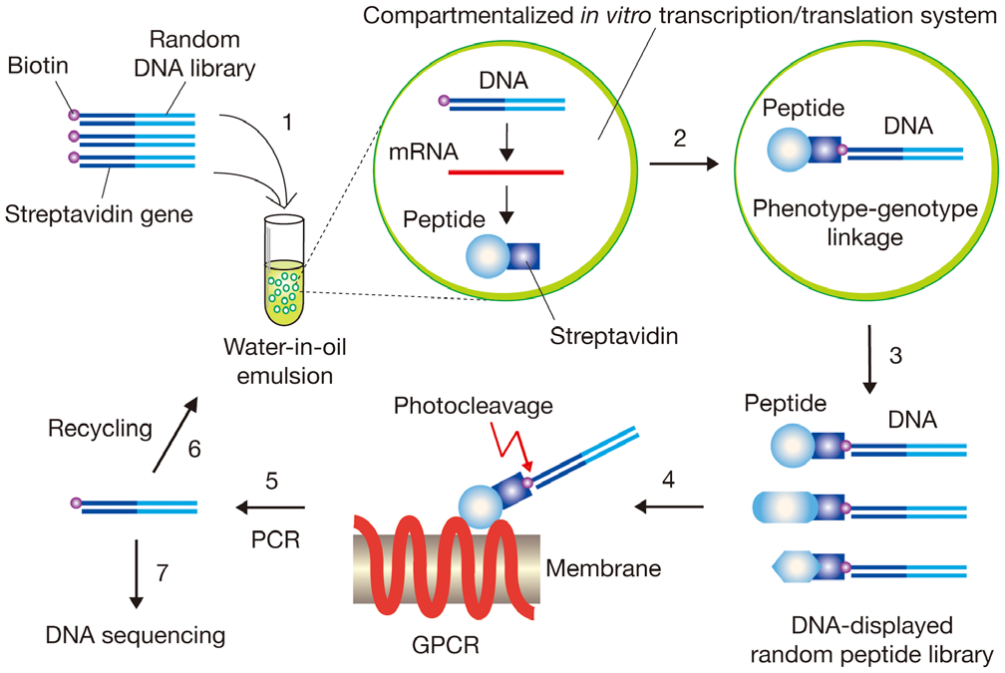
Schematic representation of DNA display selection of peptide ligands for GPCR expressed on cells. A streptavidin gene-fused random DNA library labeled with biotin through a photocleavable linker is introduced into an *in vitro* transcription/translation system (Step **1**). A single DNA molecule compartmentalized in a reversed micelle of water-in-oil emulsions is transcribed and translated *in vitro* (Step **2**). In each compartment, a translated streptavidin-fused peptide (phenotype) binds to its encoding DNA (genotype) *via* the biotin label, and a mixture of DNA-peptide conjugates is recovered from the emulsions (Step **3**). The resulting DNA-displayed random peptide library is affinity-selected with GPCR-expressing mammalian cells, and the DNA portion of binding molecules is eluted by photocleavage (Step **4**). The selected DNA is amplified by PCR (Step **5**) and used for the next round of enrichment (Step **6**) or cloning and sequencing (Step **7**).

We improved and applied the STABLE DNA display system [19]–[22] for *in vitro* selection of GPCR-ligands on whole cells (Fig. 1). In this system, the linkage of DNA (genotype) and peptide (phenotype) was accomplished in water-in-oil emulsions containing an *in vitro* transcription/translation system, in which one DNA molecule was caged in each reversed micelle on average [23]. A stable binding of streptavidin with biotin was used as the connector between DNA and its translated products [19]. The number of DNA-peptide conjugates in a library (*i.e.*, library size) is comparable with the number of emulsion droplets (10^9^–10^10^ per 1 ml of emulsion). The DNA-displayed peptide library was incubated with GPCR-expressing cells in the presence of a GRGDS pentapeptide to inhibit undesired binding of an RGD-like sequence within streptavidin to integrins on the cell surfaces [24]. We also added 0.5 M sucrose to the binding buffer to inhibit internalization of agonist peptides by receptor-mediated endocytosis [25]. Furthermore, in order to repress the large background of cell-surface proteins, glycans and lipids, the library was pre-incubated with ‘Mock’ cells without recombinant GPCR to remove nonspecific binders before incubation with the GPCR-expressing cells (not shown in Fig. 1). A Chinese hamster ovary cell line CHO-K1 is suited for this purpose, because CHO-K1 is a preferred non-human cell line for the efficient and stable expression of a variety of recombinant human proteins including GPCR [26]. Finally, we used a photocleavable 2-nitrobenzyl linker [27] between DNA and peptide for rapid and efficient recovery of selected DNA from DNA-peptide conjugates bound to GPCRs by means of simple photocleavage [22].

### Enrichment of angiotensin II genes on the hAT1R-expressing cells

**Figure 2:**
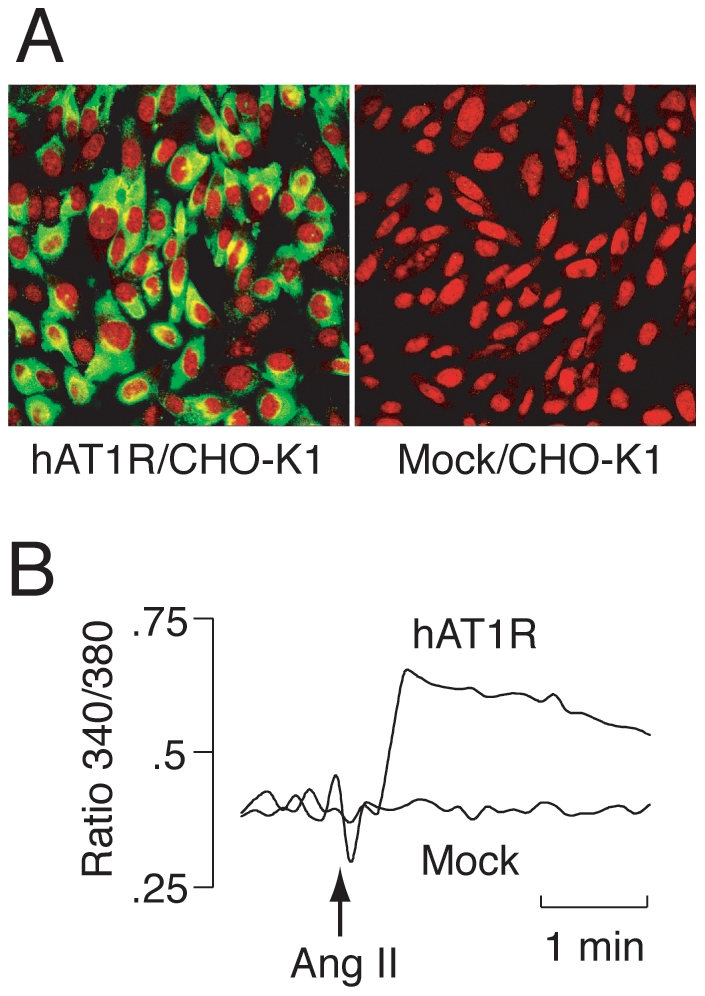
Stable expression of a model GPCR, the recombinant human angiotensin II type 1 receptor (hAT1R) with the C-terminal c-myc tag, on CHO-K1 cells. (**A**) Immunofluorescence staining of hAT1R-c-myc-expressing CHO-K1 cells (hAT1R/CHO-K1, **left**) and CHO-K1 cells transfected with the empty vector alone (Mock/CHO-K1, **right**) with anti-c-myc antibody (green). Nuclei were stained with PI (red). (**B**) Confirmation of the function of hAT1R-c-myc by monitoring changes in intracellular Ca^2+^ in response to angiotensin II (Ang II). Fura-2/AM-loaded hAT1R/CHO-K1 or Mock/CHO-K1 cells were exposed to 1 nM Ang II at the point indicated by the arrow. The results are expressed as Fura-2 fluorescence ratio (340/380 nm).

As a model GPCR and its ligand, the human angiotensin II type 1 receptor (hAT1R) and octapeptide angiotensin II (Ang II; Asp^1^-Arg^2^-Val^3^-Tyr^4^-Ile^5^-His^6^-Pro^7^-Phe^8^) were used here. Stable expression of recombinant hAT1R with a C-terminal c-myc tag on CHO-K1 cells was confirmed by immunostaining with anti-c-myc antibody (Fig. 2A). The binding of Ang II to the hAT1R/CHO-K1 cells was confirmed by radio ligand binding assay: the dissociation constant and the number of ligand binding sites were estimated as *K*_d_ = 8.2 nM and 8.3×10^5^ binding sites/cell, respectively (data not shown). In addition, the function of the recombinant hAT1R was confirmed by monitoring the change in intracellular calcium in response to Ang II (Fig. 2B). These results indicate that a sufficient amount of the model GPCR, hAT1R, was expressed on cell membranes in active form(s), and thus, hAT1R can serve as a bait receptor protein for further *in vitro* selection experiments.

To confirm whether not only Ang II peptides, but also streptavidin-fused Ang II proteins can bind to hAT1R, streptavidin-Ang II genes with the generally-used linkers were *in vitro* transcribed and translated, and the products were incubated with the hAT1R/CHO-K1 cells. It was found that the peptide fused with streptavidin through a helical linker [28] (four repeats of ‘GAAAK’) efficiently and specifically bound to the hAT1R/CHO-K1 cells, while the fusion protein with a Gly-rich flexible linker (five repeats of ‘SGGGG’) nonspecifically bound to both the hAT1R/CHO-K1 and the Mock CHO-K1 cells, and the fusion protein with no linker could not bind to both cells (data not shown), perhaps due to steric hindrance. The results indicate that a peptide ligand can specifically bind to its receptor expressed on CHO-K1 cells even if the large streptavidin tetramer is fused with the peptide through the helical linker.

**Figure 3:**
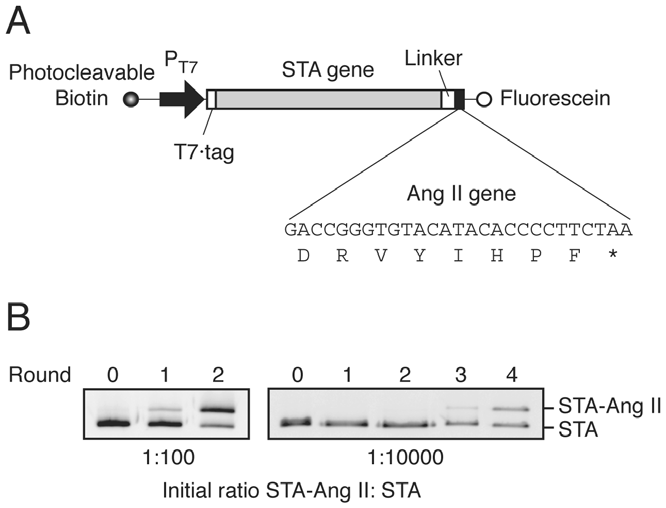
Construction and enrichment of the streptavidin-fused angiotensin II (STA-Ang II) gene in multiple rounds of DNA-display selection on hAT1R/CHO-K1 cells. (**A**) A schematic representation of the DNA template for *in vitro* transcription/translation. DNA was labeled during PCR with photocleavable biotin [22] at the upstream ends and with fluorescein at the downstream ends, using labeled primers. The translated open reading frame consists of sequences for a T7·tag, streptavidin (STA), a peptide linker, and Ang II gene. The 5′-UTR fragment contains T7 promoter. (**B**) Reaction mixtures containing 1∶100 or 1∶10,000 molar ratio of STA-Ang II: STA genes were emulsified. The DNA after each round of selection was PCR-amplified with a fluorescein-labeled primer and analyzed by 15% PAGE with an imaging analyzer.

By using the hAT1R-expressing cells as bait, we demonstrated that genes encoding streptavidin-fused angiotensin II (termed STA-Ang II) could be selected from at least a 10^4^-fold excess of genes encoding streptavidin without Ang II (termed STA) using the improved DNA display. The STA-Ang II and STA genes were mixed in a ratio of 1∶100 or 1∶10,000, and then subjected to several rounds of the DNA display selection procedure using water-in-oil emulsions (Fig. 1). Two rounds of selection of the 1∶100 ratio of STA-Ang II: STA genes and four rounds of selection of the 1∶10,000 ratio each resulted in a roughly 1∶1 final gene ratio, indicating an enrichment factor of about 10-fold per round (Fig. 3). This enrichment efficiency is comparable with that in our previous studies using bait-immobilized beads [19], [20].

### *In vitro* selection of randomized peptide libraries

**Figure 4:**
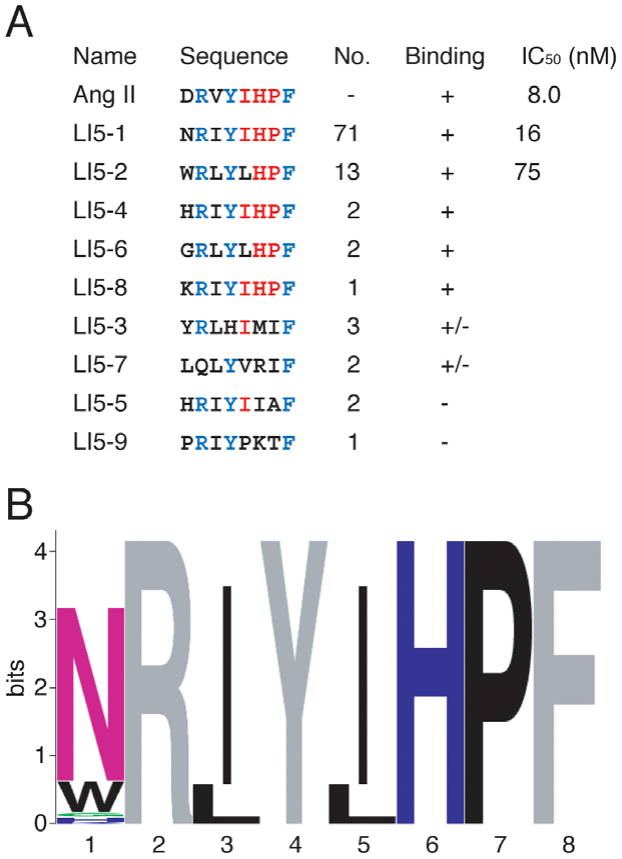
Amino acid sequences of selected clones from the randomized library-I. (**A**) The designed sequence is XRΩYΩXΩF where X = F, L, I, M, V, S, P, T, A, Y, H, Q, N, K, D, E, C, W, R or G; and Ω = F, L, I, M, V, S, P, T or A. Fixed residues are shown in blue, and conserved residues are red. The number of clones containing each sequence is indicated. Sequences of seven clones with a frameshift in the preceding linker region are not shown. The binding activity (+, specific binding; +/−, nonspecific binding; −, no binding) of each peptide is also shown in Figures 6 and 7. IC_50_ was determined by means of competitive binding assays at various concentrations of synthetic peptides (see MATERIALS AND METHODS and Figure S1). (**B**) Sequence logos representation [45], [46] of the peptide sequences with specific binding activity (+). The height of each column reflects the bias from random of particular residues. Fixed residues are shown in gray. Polar amino acids containing an amide group (Q, N) and the rest of them are shown in purple and green, respectively, basic charged residues in deep blue, and hydrophobic residues in black.

Next, we applied the DNA display method to selection of randomized peptide libraries on whole cells. Since early studies using synthetic analogues of Ang II revealed that Arg^2^, Tyr^4^ and Phe^8^ in Ang II directly interact with hAT1R [29]–[31], the three residues were fixed in the randomized Ang II library-I. The DNA-displayed randomized peptide library-I was captured on the hAT1R/CHO-K1 cells, which were then washed, and exposed to UV irradiation for elution. After five rounds of selection, DNA was PCR-amplified and cloned. Randomly chosen clones were analyzed by DNA sequencing, and the binding activity of each distinct, in-frame clone was further confirmed (see next section for details). Consequently, five Ang II-like sequences were obtained (Fig. 4A, Binding, +). Not only fixed residues Arg^2^, Tyr^4^ and Phe^8^, but also the residues His^6^ and Pro^7^ of the original Ang II sequence were conserved at frequencies of 100% (Fig. 4B). Other residues Asp^1^ and Val^3^ did not appear (0%) in the selected clones, while Ile^5^ was also highly conserved (Fig. 4B).

**Figure 5:**
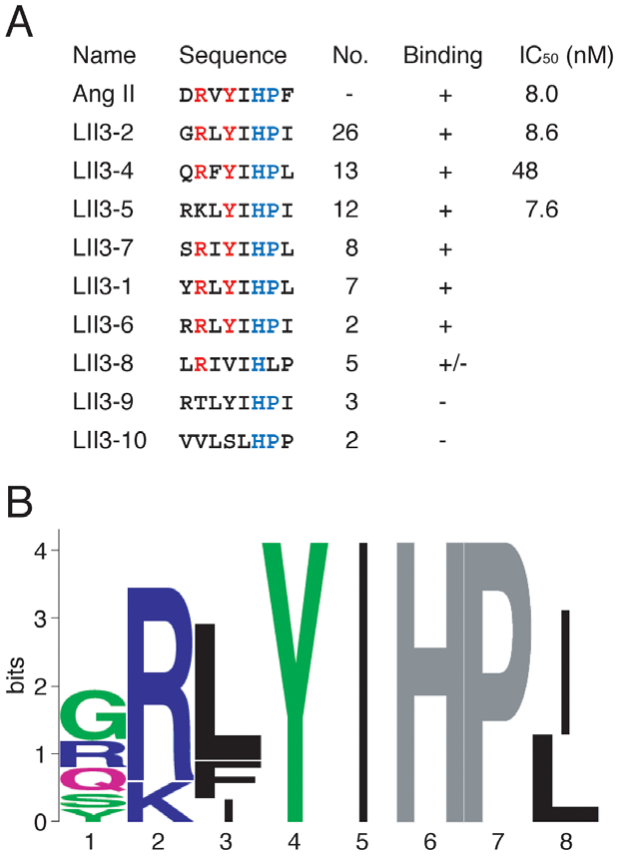
Amino acid sequences of selected clones from the randomized library-II. (**A**) The designed sequence is XXΨXΘHPΩ where X = F, L, I, M, V, S, P, T, A, Y, H, Q, N, K, D, E, C, W, R or G; Ψ = F, L, I or V; Θ = I or L; and Ω = F, L, I, M, V, S, P, T or A. Sequences of four clones (including LII3-3) with a frameshift in the preceding linker region are not shown. Fixed residues are shown in blue, and conserved residues are red. (**B**) Sequence logos representation. For details, see the legend of Figure 4.

To investigate whether the residues Arg^2^, Tyr^4^ and Phe^8^ in Ang II fixed in the library-I are truly essential for binding to hAT1R, we further constructed and screened the randomized Ang II library-II, in which the 2nd, 4th and 8th positions were randomized, while His^6^ and Pro^7^ were fixed. The sequencing and binding analyses revealed that the residues Arg^2^ and Tyr^4^ were conserved at frequencies of 80 and 100%, respectively, while Phe^8^ did not appear (0%) in the selected clones (Fig. 5B).

### Characterization of the selected peptides

**Figure 6:**
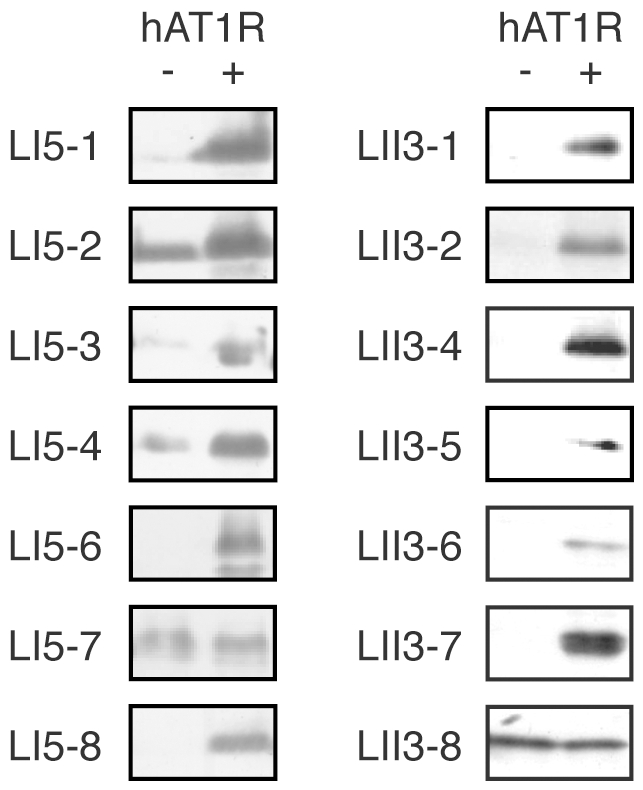
Binding assays of selected peptides from the randomized libraries with the hAT1R-expressing cells. Selected streptavidin-fused peptides were generated by the PURE system. An aliquot of the reaction mixture was incubated with the hAT1R/CHO-K1 cells (hAT1R +) or the Mock/CHO-K1 cells (hAT1R −). The cells were washed, and the bound molecules were detected with anti-T7·tag antibody. The sequences of the peptides are shown in Figures 4A and 5A.

As mentioned above, we first investigated the binding activities of streptavidin-fused peptides selected from the library-I (Fig. 4A) and the library-II (Fig. 5A). Each streptavidin-fused peptide was produced independently and captured on the hAT1R-expressing CHO-K1 cells. Then the cells were washed, and residual proteins were detected by Western blotting. As shown in Figure 6, 12 out of 18 peptides specifically bound to hAT1R/CHO-K1 cells. LI5-7 and LII3-8 nonspecifically bound to the Mock/CHO-K1 cells with the empty vector alone, as well as the hAT1R/CHO-K1 cells. LI5-5, LI5-9, LII3-9 and LII3-10 (not shown) did not bind to hAT1R/CHO-K1. Although nonspecific binding to the Mock/CHO-K1 cells was also seen for LI5-1 to LI5-4, especially for LI5-2, each band for the Mock/CHO-K1 cells is weaker than that for hAT1R/CHO-K1 cells, respectively, and thus they were served as candidates for further characterization.

**Figure 7:**
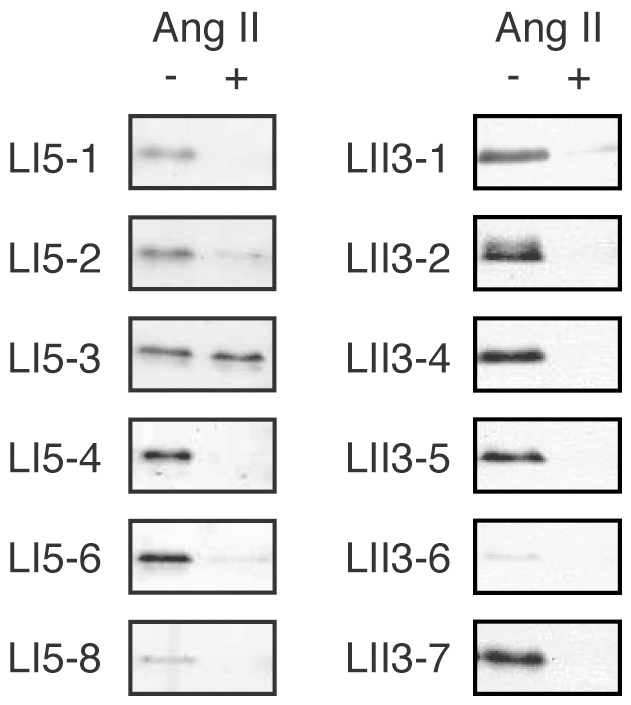
Competitive binding assays. Selected streptavidin-fused peptides were incubated with the hAT1R/CHO-K1 cells in the presence (+) or the absence (−) of the free Ang II peptide (100 nM) as a competitor. For details, see the legend of Figure 6 and MATERIALS AND METHODS.

To clarify whether the selected 12 peptides bind to hAT1R in the same manner as Ang II, we next performed competitive binding assays. Except for LI5-3, the peptides bound to hAT1R/CHO-K1 cells only in the absence of the Ang II competitor (Fig. 7), indicating that the selected peptides do interact with hAT1R at the Ang II-binding site. Again, some content of LI5-2 non-specifically bound to the cell-surface (Fig. 6) and thereby seems not to be completely competed out by Ang II (Fig. 7). A reason may be a relatively higher hydrophobicity of LI5-2 peptide with Trp^1^ in comparison with other peptides having hydrophilic amino acids such as Asn, His or Arg at the position 1.

Furthermore, IC_50_ values were determined by means of competitive binding assays at various concentrations of synthetic peptides (Fig. S1) for the frequent clones LI5-1 (71 clones) and LI5-2 (13 clones), as shown in Figure 4A, as well as LII3-2 (26 clones), LII3-4 (13 clones) and LII3-5 (12 clones), shown in Figure 5A.

**Figure 8:**
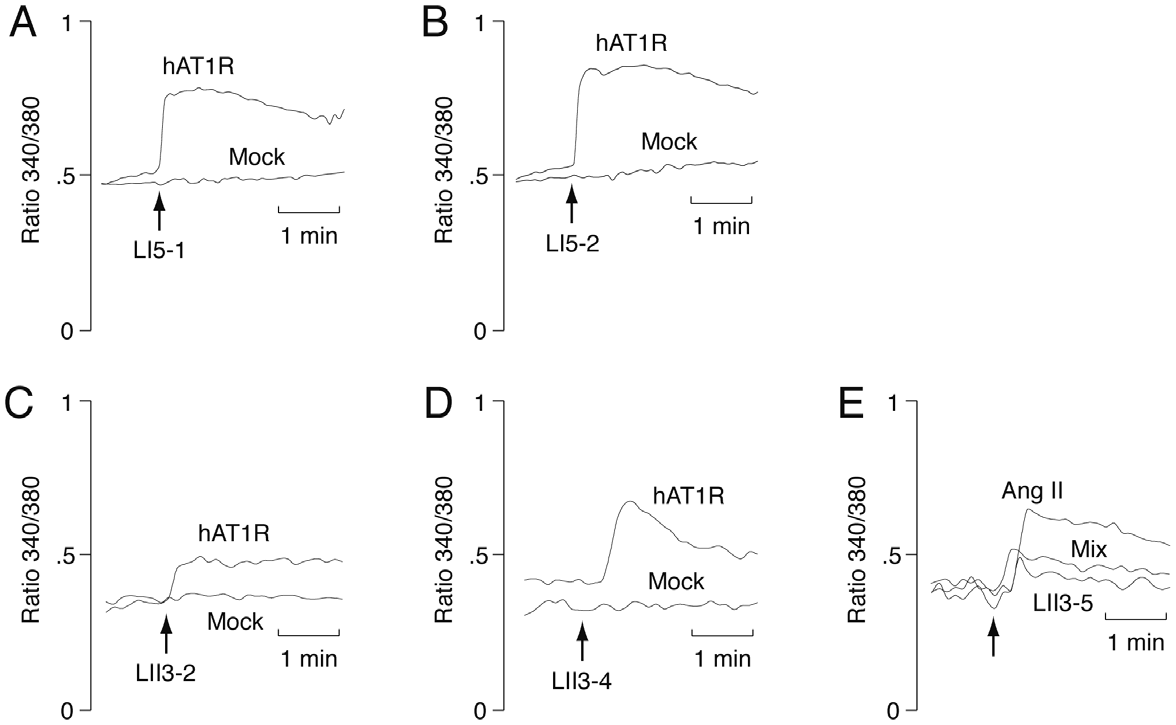
Effect of selected peptides on the concentration of calcium in the hAT1R-expressing cells. Fura-2/AM-loaded hAT1R/CHO-K1 or Mock/CHO-K1 cells were exposed to synthetic octapeptides (**A**) LI5-1 (10 nM), (**B**) LI5-2 (10 nM), (**C**) LII3-2 (10 nM), (**D**) LII3-4 (100 nM), and (**E**) LII3-5 (1 µM) and/or Ang II (1 nM), respectively, as indicated by the arrows. The results are expressed as Fura-2 fluorescence ratio (340/380 nm).

Finally, we confirmed the biological activity of selected peptides by calcium imaging (Fig. 8). The concentration of calcium in the hAT1R-expressing cells increased in response to LI5-1, LI5-2, LII3-2 and LII3-4, indicating that these peptides have agonist activity, like Ang II (Fig. 8A-D). On the other hand, LII3-5 did not enhance the calcium concentration, but inhibited the effect of Ang II (Fig. 8E), indicating that this peptide has antagonist activity.

## Discussion

In this study, completely *in vitro* selection of peptide ligands for a full-length GPCR expressed on whole cells was accomplished by using an *in vitro* DNA display technology. With Ang II receptor as a model GPCR, wild-type and mutated Ang II peptides were successfully selected from model libraries and randomized peptide libraries, respectively. As shown in Fig. 3, when only Ang II was the binding sequence in the doped library, the Ang II sequence was selected from the model library. However, no wild-type sequence was selected from the randomized libraries, though a variety of Ang II-like peptides were identified (Figures 4 and 5). The constrained random libraries contain a variety of binding sequences, some of which become fixed by random genetic drift during the iterative selection process. Thus, it is not unusual for selection experiments (e.g., Refs. [9]–[13]) to be failed to pick up native peptide sequences from partially or completely randomized peptide libraries.

The analysis of the selected peptides revealed that the residues Arg^2^, Tyr^4^, His^6^ and Pro^7^ in Ang II are important for binding to hAT1R. This result is consistent with the previous finding that the binding affinity of synthetic Ang II to hAT1R was affected by Ala substitution at positions 2, 4, 6 and 7, respectively (*K*_d_>10 µM) [32]. However, LII3-5 with Lys^2^ retains affinity as high as that of wild-type Ang II (Fig. 5A), which is inconsistent with the previous study [33]: the affinity of [Lys^2^]Ang II for rat AT1R was lower than that of wild-type Ang II.

While the residue Phe^8^ is important for high agonist activity [29], [31], a series of Ang II analogs mutated at the C-terminal position 8 (*e.g.*, Ile^8^ and Leu^8^) exhibit antagonist activity [34], [35]. However, the selected peptides LII3-2 with Ile^8^ and LII3-4 with Leu^8^ possess agonist activity (Fig. 8C,D), indicating that nonconserved positions 1 and 3 are also important in determining the function of Ang II-like peptides. Furthermore, the shapes of the calcium fluxes for the mutated peptides are somewhat different from that for wild-type Ang II. It may reflect a possible different signaling by the selected peptides, because there is a growing body of literature suggesting that different ligands bind to and activate the same GPCR through different signaling pathways [36], [37]. Thus, further studies for selection of peptide ligands have the potential to contribute to the GPCR biology in the future.

The use of DNA display for searching peptide ligands offers several advantages over other display technologies, such as phage display and mRNA display. First, as mentioned in the introduction, the size of DNA display libraries based on cell-free protein synthesis is usually greater than that of phage libraries using bacterial expression, and the chemical stability of DNA permits selection targeting full-length GPCRs expressed on cell-surface, where RNA would be easily degraded. Further, each peptide in the conventional phage display and mRNA display libraries should be linked with its genotype at the C-terminus, while a peptide can be fused with both N- and C-terminals of streptavidin in DNA display [38]. The free N- or C-terminus of peptide ligands is often important for their biological activities. Finally, the use of the reconstituted transcription/translation system in DNA display allows various applications; for example, the incorporation of unnatural amino acids [39], [40] for *in vitro* selection of peptide mimetic compounds. Thus, the method we describe should prove useful not only for the mutation analysis of ligand/receptor interaction, but also for screening of agonists and antagonists for disease-related GPCRs that can be expressed functionally on the surface of CHO-K1 cells, and for the identification of peptide and protein ligands for orphan GPCRs except for those with non-peptide endogenous ligands.

## Materials and Methods

### Preparation of GPCR-expressing mammalian cells

The hAT1R gene (1080 bp) [41] was amplified from a human liver cDNA library (from Prof. J.-I. Inoue; Institute of Medical Science, University of Tokyo) by means of PCR with *Ex Taq* DNA polymerase (Takara Shuzo) using the primers hAT1R-F and hAT1R-R (all primer sequences used in this study are listed in Table S1). The PCR product was digested with EcoRI and XbaI and then cloned into the identical restriction-enzyme sites of a vector pEF1/myc-HisA (Invitrogen) comprising an EF1α promoter and fusion-tag sequences encoding C-terminal c-myc and polyhistidine tags. The resulting plasmid pEF1-hAT1R-MycHis was confirmed with an ABI PRISM 3100 genetic analyzer (Applied Biosystems), purified with an endofree plasmid maxi kit (Qiagen), and used for transformation of the mammalian cell line CHO-K1 [a Chinese hamster ovary cell line purchased from RIKEN Cell Bank (Ibaraki, Japan) in 2000] with lipofectamine 2000 (Invitrogen). As a control, CHO-K1 cells were also transfected with the empty vector alone (Mock/CHO-K1). The transfected cell lines were kept under selection pressure of 400 µg/ml G418. The expression of the recombinant hAT1R was confirmed by Western blot analysis of cell lysates using mouse anti-c-myc monoclonal antibody (MBL) and an ECL plus Western blotting analysis system (Amersham Biosciences).

### Immunofluorescence staining

The hAT1R-expressing mammalian cells were plated on a micro cover glass sunk in complete medium [10% (v/v) FBS, 90% (v/v) Ham's F-12, 100 units/ml penicillin, 100 µg/ml streptomycin, 400 µg/ml G418] and incubated for 48 h at 37°C in an atmosphere of 5% CO_2_ in air. Prior to immunostaining, cells were fixed in PBST with 3.7% paraformaldehyde for 10 min at room temperature, treated with 0.1 mg/ml RNase A (DNase-free; Invitrogen) for 20 min at 37°C, and blocked in PBS with 1% BSA for 30 min at room temperature. Antibodies were diluted in PBS with 1% BSA. Immunofluorescence staining was performed with mouse anti-c-myc monoclonal antibody (Santa Cruz Biotechnology) for 45 min at room temperature. The cover glass was washed three times with PBS, incubated with goat anti-mouse Alexa Fluor 488-conjugated antibody (Molecular Probes) for 50 min and washed three times with PBS. For nuclear staining, the cover glass was incubated with 500 nM propidium iodide (PI; Molecular Probes) in 2× SSC (0.3 M NaCl, 30 mM sodium citrate, pH 7.0) for 5 min at room temperature and washed three times with 2× SSC. Then the cover glass was inverted and set on a slide glass. The slides were viewed with a confocal microscope (Nikon Eclipse E600 microscope and Bio-Rad Radiance 2000 scanning system) for Alexa Fluor 488 (495/519 nm) and PI (536/617 nm).

### Radio ligand binding assays

Cells were harvested in 48-well plates (IWAKI) at a density of approximately 6×10^5^ cells/well. The cells were blocked with Ham's F12 medium containing 1% BSA, then [^125^I]Sar^1^,Ile^8^-Ang II was added (2,000 Ci/mmol; Amersham Biosciences; final concentration in 150 µl final volume ranging from 0.05 nM to 20 nM). After incubation for 30 min at room temperature, the cells were washed three times with 500 µl of PBS containing 1% BSA and radioactivity was counted with a MINAXI auto-gamma 5000 scintillation counter (Canberra-Packard). Values for receptor capacity and affinity were obtained by Scatchard analysis.

### Calcium imaging

The intracellular concentration of calcium [Ca^2+^]_i_ was measured by incubating hAT1R-expressing cells with the fluorescent Ca^2+^ indicator, 1-[6-amino-2-(5-carboxy-2-oxazolyl)-5-benzofuranyloxy]-2-(2-amino-5-methylphenoxy)ethane-*N*,*N*,*N*′,*N*′-tetraacetic acid penta-acetoxymethyl ester (Fura-2/AM) as previously described [42]. Briefly, the cells in fresh BSS (balanced salt solution; 20 mM HEPES, pH 7.3, 130 mM NaCl, 5.4 mM KCl, 5.5 mM d-glucose, 1.8 mM CaCl_2_ and 0.8 mM MgSO_4_) were loaded at 37°C for 30 min with 5 µM Fura-2/AM, conditioned in flowing BSS at 37°C, then exposed to the stimulating reagent Ang II or synthetic peptides for selected clones by changing the BSS flow to peptide-containing BSS flow for 30 s. The fluorescence of the cells was then measured at the emission wavelength of 510 nm with sequential excitation at 340 nm and 380 nm. The 340/380 ratio can be converted to [Ca^2+^]_i_ according to the formula presented by Grynkiewicz *et al.* [43].

### Preparation of DNA libraries

Two libraries of streptavidin-fused random peptides were amplified by three steps of PCR from a pSta4-derived plasmid [20] carrying a streptavidin gene with the N-terminal T7·tag and the C-terminal helical peptide linker by the 1st step PCR with *Ex Taq* DNA polymerase, using forward primer T7tagF-M and reverse primer HL4-SG-RYF-NYN-R (for library-I) or HL4-SG-MTH-NTN-NYN-R (for library-II) (Table S1). Each PCR product was re-amplified using forward primer T7F-M and reverse primer T7R-M (library-I) or T7R-M2 (library-II), and finally re-amplified using photocleavable biotin (PCB)-labeled T7F and T7R primers. The PCR products were purified with a QIAquick PCR purification kit (Qiagen). Similarly, an expressible DNA fragment encoding a streptavidin-Ang II fusion protein (a positive control) and a streptavidin gene without Ang II (a negative control) were amplified by three-step PCR using reverse primer HL4-AT2-R or HL4-R, respectively, in the 1st step. All PCR programs consisted of 15–25 cycles of denaturation at 98°C for 10 s, annealing at 60°C for 30 s, and extension at 72°C for 1 min.

### Preparation of DNA-displayed peptide libraries

*In vitro* transcription/translation reactions in water-in-oil emulsions were performed as previously described [19], [23] with the following modifications. The PURE system classic II kit (Post Genome Institute Co., Ltd.) based on a reconstituted *E. coli* transcription/translation system [44] was used as the water phase, and mineral oil (Nacalai Tesque) containing 4.5% (v/v) Span85 (Nacalai Tesque) and 0.5% (v/v) Tween80 (Nacalai Tesque) was used as the oil phase. The DNA concentration was 50 pM in each round of selection except for the first round, in which it was 200 pM, and the fifth round, in which it was 10 pM. The amounts of emulsion were 10 ml (including 500 µl of PURE system) for the first round, 4 ml (200 µl) for the second round, 2 ml (100 µl) for the third round, and 1 ml (50 µl) for further rounds. Thus, the numbers of DNA molecules in each library were ∼6×10^10^ for the first round, ∼6×10^9^ for the second round, ∼3×10^9^ for the third round, ∼2×10^9^ for the fourth round, and ∼3×10^8^ for the fifth round. The emulsions were incubated for 2 h at 37°C. To recover the water phase, the emulsions (1 ml) were spun at 2,000 *g* and the supernatant (750 µl) was mixed with 200 µl of quenching buffer [Hanks' balanced salt solution (HBSS) containing 0.5 M sucrose, 1% (w/v) BSA, 1% protease inhibitor cocktail (Nacalai Tesque), 100 µM GRGDS peptide (Peptide Institute, Inc.), 1 mg/ml salmon sperm DNA (Stratagene), 1 µM biotin] and then centrifuged at 2,000 *g* for 5 min. The water phase was recovered and 1 ml of water-saturated diisopropyl ether was added. The mixture was inverted 20 times and centrifuged at 19,000 *g* for 5 min, and the ether phase was removed. The water phase was exposed to a vacuum to remove residual diisopropyl ether. The resulting DNA-displayed peptide library was used for affinity selection.

### Affinity selection on whole cells

Binding and selection experiments with DNA-displayed peptides for receptors on whole cells were usually carried out in 1 ml of HBSS containing 0.5 M sucrose, 1% BSA, 1% protease inhibitor cocktail, 100 µM GRGDS peptide, and 1 mg/ml salmon sperm DNA at room temperature with gentle shaking for 1 h. The library was pre-incubated with Mock cells without recombinant hAT1R receptor to remove nonspecific binders and then incubated with the hAT1R-expressing cells. The cells were washed several times with HBSS containing 0.5 M sucrose, 0.1% protease inhibitor cocktail, 10 µM GRGDS peptide and 10 µg/ml salmon sperm DNA. Finally 1 ml of HBSS with 0.5 M sucrose was added and the selected DNA was eluted by exposure to UV irradiation from a Black Ray XX-15 UV lamp (Ultraviolet Products Inc.) at a distance of 15 cm (emission peak 365 nm, 300 nm cut-off, 1.1 mW intensity at 31 cm) for 15 min in a cold room [22]. The eluted DNA was purified by ethanol precipitation and amplified by PCR with KOD-plus DNA polymerase (Toyobo) using PCB-labeled T7F forward primer and T7R-M (for library-I) or T7R-M2 (for library-II) reverse primer (25–35 cycles of 94°C, 15 s; 62°C, 30 s; and 68°C, 60 s). The PCR products were electrophoresed on 1% agarose gel, purified with a Recochip (Takara Shuzo) and the QIAquick PCR purification kit, and used as template DNA described in the previous section “Preparation of DNA-displayed peptide libraries” for the next round of selection. After several rounds of the library preparation and affinity selection, to identify selected peptides, DNA was amplified with non-labeled primers, cloned with a TOPO TA cloning kit (Invitrogen), and sequenced with the ABI PRISM 3100 genetic analyzer using Sta150F or Sta75F primer. Sequence logos representation [45], [46] of amino-acid sequences was created with WebLogo Version 2.8.2 (http://weblogo.berkeley.edu/).

### Streptavidin-fused peptide binding assays

Streptavidin-fused peptides for selected clones were prepared separately by using the PURE system classic II kit with 20 nM DNA for 1 h at 37°C. The streptavidin-fused peptides were incubated with the hAT1R-expressing cells for 10 min at room temperature in the presence 0–200 nM synthetic peptides (see next section) as competitors, and washed as described in the previous section. Then the binding proteins were recovered using 0.5 ml of lysis buffer (1% protease inhibitor cocktail and 4 M urea) on ice for 30 min. The cell lysates were analyzed by 15% SDS-PAGE and Western blot analysis using anti-T7·tag antibody (Novagen) followed by HRP-conjugated secondary antibody (Chemicon). The streptavidin-fused peptides with T7·tag were quantitatively detected by an ECL chemiluminescence kit and Hyperfilm ECL (GE Healthcare) [47].

### Peptide Synthesis

Peptides of the LI5-series were synthesized by Peptide Institute Inc. Peptides of the LII3-series were synthesized with an automated solid-phase peptide synthesizer (model PSSM-8, Shimadzu). The peptides were purified by reversed-phase high-performance liquid chromatography (C_18_ column, 250 mm×20 mm inside diameter) with a linear gradient of water containing 0.1% trifluoroacetic acid (TFA) and acetonitrile containing 0.1% TFA at a flow rate of 10 ml/min. The major fractions were lyophilized, and the peptides were characterized by matrix-assisted laser desorption ionization time-of-flight mass spectrometry (MALDI-TOF MS) (Autoflex, Bruker Daltonics).

## Supporting Information

Figure S1 **Competition curves for selected peptides.** Streptavidin-fused Ang II was prepared with the PURE system, incubated with the hAT1R/CHO-K1 cells in the presence of various concentrations of synthetic peptides LI5-1 (filled circles), LI5-2 (filled squares), LII3-2 (open circles), LII3-4 (open squares) and LII3-5 (open triangles), washed and analyzed by 15% SDS-PAGE and Western blot analysis. For details, see MATERIALS AND METHODS.
(PDF)

Table S1 **Oligonucleotide sequences.** N = A, C, G or T; D = A, G or T; M = A or C; R = A or G; K = G or T. T7F labeled with photocleavable biotin (PCB) and T7R labeled with fluorescein were used for preparation of labeled DNAs.
(DOC)
