# Supplementary figures and images for: DNA Display Selection of Peptide Ligands for a Full-Length Human G Protein-Coupled Receptor on CHO-K1 Cells
Source: PLoS One. 2012 Jan 10;7(1):e30084. doi: 10.1371/journal.pone.0030084 (PMC3254644; doi:10.1371/journal.pone.0030084)

Relative Binding (%)

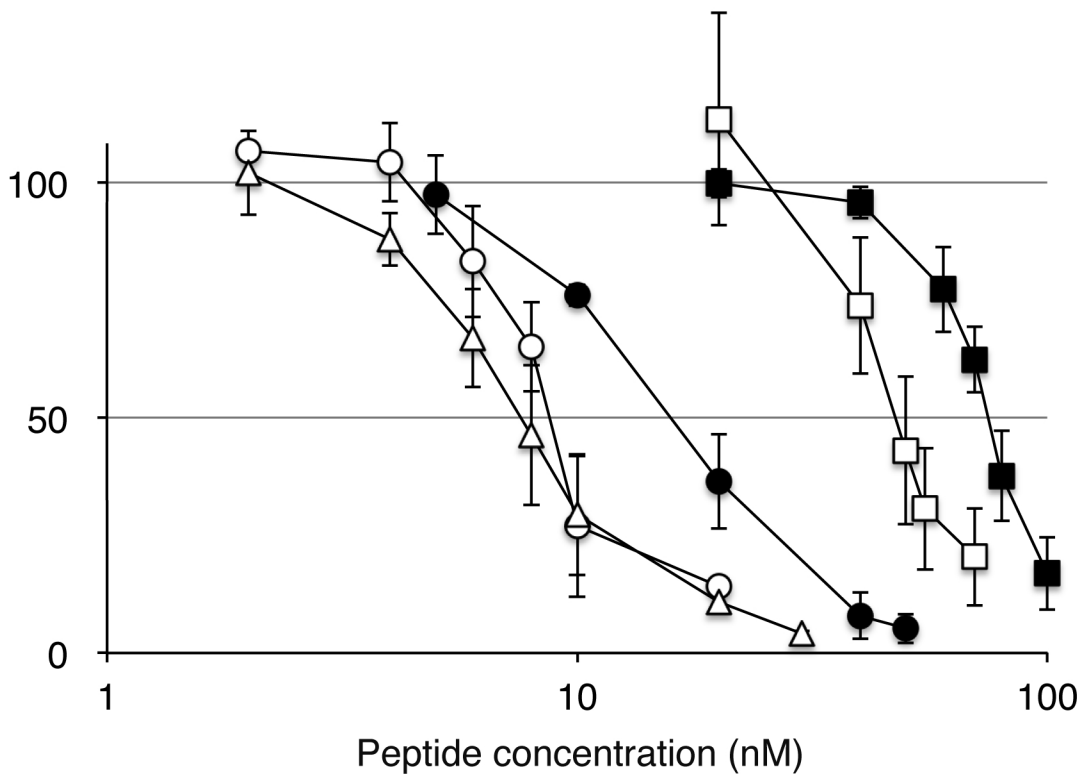

Supplement: Figure S1 — Competition curves for selected peptides. Streptavidin-fused Ang II was prepared with the PURE system, incubated with the hAT1R/CHO-K1 cells in the presence of various concentrations of synthetic peptides LI5-1 (filled circles), LI5-2 (filled squares), LII3-2 (open circles), LII3-4 (open squares) and LII3-5 (open triangles), washed and analyzed by 15% SDS-PAGE and Western blot analysis. For details, see MATERIALS AND METHODS. (PDF) [file pone.0030084.s001.pdf]
