# Supplementary material for: DNA Display Selection of Peptide Ligands for a Full-Length Human G Protein-Coupled Receptor on CHO-K1 Cells
Source: PLoS One. 2012 Jan 10;7(1):e30084. doi: 10.1371/journal.pone.0030084 (PMC3254644; doi:10.1371/journal.pone.0030084)
Supplement: Table S1 — Oligonucleotide sequences. N = A, C, G or T; D = A, G or T; M = A or C; R = A or G; K = G or T. T7F labeled with photocleavable biotin (PCB) and T7R labeled with fluorescein were used for preparation of labeled DNAs. (DOC) [file pone.0030084.s002.doc]

| Oligonucleotide | Sequence |
| --- | --- |
| hAT1R-F | 5'-GGAATTCGCCACCATGATTCTCAACTCTTCTACTGAAGATG-3' |
| hAT1R-R | 5'-CCGTCTAGATAACTCAACCTCAAAACATGGTGC-3' |
| HL4-AT2-R | 5'-GCTAGTTATTGCTCAGCGGTTAGAAGGGGTGTATGTACACCCGGTCGCCACCTCCACCCGATCGAGC-3' |
| HL4-R | 5'-GCTAGTTATTGCTCAGCGGTTAAGCTTTAGCTGCTGCCTCTTTAGCGGCTGCCTCCTTAGCCGCCGCTTC-3' |
| HL4-SG-RYF-NYN-R | 5'-CTCAGCTTCCTTTCGGGCTTTGTATTCATTAGAANRNMNNNRNGTANRNCCGMNNGCCACCTCCACCCGATCGAGCTTTAGCTGCTGCCTC-3' |
| HL4-SG-MTH-NTN-NYN-R | 5'-CTCAGCTTCCTTTCGGGCTTTCTATTCAATTANRNCGGATGDAKMNNNANMNNMNNGCCACCTCCACCCGATCGAGCTTTAGCTGCTGCCTC-3' |
| Sta150F | 5'-GCAACCCGCTGGACGCTGTTC-3' |
| Sta75F | 5'-TGGACTGTTGCTTGGAAGAAT-3' |
| T7F | 5'-CGGCATATGATCCCGCGAAATTAATACG-3' |
| T7F-M | 5'-CGGCATATGATCCCGCGAAATTAATACGACTCACTATAGGGAGACCACAACGGTTTCCCTCTAGAAATAATTTTGTTTAACTTTAAGAAG-3' |
| T7R | 5'-GCTAGTTATTGCTCAGCGG-3' |
| T7R-M | 5'-GCTAGTTATTGCTCAGCGGTGGCAGCAGCCAACTCAGCTTCCTTTCGGGCTTTGTATTCATTA-3' |
| T7R-M2 | 5'-CTAGTTATTGCTCAGCGGTGGCAGCAGCCAACTCAGCTTCCTTTCGGGCTTTCTATTCAATTA-3' |
| T7tagF-M | 5'-CAACGGTTTCCCTCTAGAAATAATTTTGTTTAACTTTAAGAAGGAGATGCCACCATGGCTAGCATGACTGGTGGACAGC-3' |
